# Editorial: With the Eyes on Non-coding RNAs

**DOI:** 10.3389/fcell.2021.737703

**Published:** 2021-07-29

**Authors:** Ruth Ashery-Padan, Brian D. Perkins, Ivan Conte

**Affiliations:** ^1^Department of Human Molecular Genetics and Biochemistry, Sackler Faculty of Medicine, Sagol School of Neuroscience, Tel-Aviv University, Tel-Aviv, Israel; ^2^Department of Ophthalmic Research, Cole Eye Institute, Cleveland Clinic, Cleveland, OH, United States; ^3^Department of Biology, University of Naples Federico II, Naples, Italy; ^4^Telethon Institute of Genetics and Medicine, Pozzuoli, Italy

**Keywords:** lncRNA, miRNAs, retina, visual system, eye

Non-coding RNAs (ncRNAs) are a class of structural and regulatory RNAs that have received increasing attention because of their implication in the disease development and a variety of physiological and pathological processes. These includes tissue-specific and “housekeeping” ncRNAs, including long non-coding RNAs, microRNAs, circular-RNA, natural antisense transcripts (NATs), and several other poorly characterized ncRNAs that regulate gene expression in a variety of ways at epigenetic, chromatin remodeling, transcriptional, and translational levels. Regulatory ncRNAs exhibit dynamic temporal and spatial expression profiles in specific cellular contexts and contribute to tissue patterning and to the control of different cellular programs, including proliferation, differentiation, apoptosis, migration, and invasion. The 12 selected papers in this special Research Topic entitled “With the Eyes on Non-Coding RNAs” highlights the significant scientific breakthroughs on the physiological and pathological role of ncRNAs, offering additional insights into the challenges in understanding the functions of ncRNAs in the eye and improve the diagnostic ability, making them as targets of future novel therapies to treat ocular diseases.

The most compelling evidence for the contribution of individual miRNAs to photoreceptor cell homeostasis and functions has been well-documented by Pawlick et al. They summarize recent advances in how single miRNA can regulate hundreds of mRNAs, highlights the emerging contribution of miR-124 and the miR-183/96/182 cluster in photoreceptors in health and disease. They also discuss the experimental validation and manipulation approaches to study complex miRNA/mRNA regulatory networks in several animal models.

Focusing on the role of regulatory ncRNAs (e.g., miRNAs, lncRNAs, circular RNAs, and antisense RNAs), Carrella et al. examine emerging novel regulation and functions of these classes of ncRNAs in conferring robustness to photoreceptor development and function. First, this review examines the evidence for the role of miRNAs to photoreceptor pathophysiology and discusses the role of specific lncRNAs and circRNAs in targeting miRNAs for the proper control of photoreceptor homeostasis and function. Moreover, Carrella et al. further summarizes the current knowledge on miRNAs involvement in mitochondrial eye diseases and delve on the therapeutic implications including identification of miRNAs as biomarkers and for treatment of retinal pathologies.

Importantly, in this topic Konar et al. gave an outstanding overview about the role of specific miRNAs in the control of Müller Glia reprogramming during retina regeneration as well as putative future therapeutic targets for treatment of visual disorders and damage. In addition, they discuss about main model systems and technologies that are used to evaluate miRNAs in the retina, along with their advantages and limitations.

miRNAs' function in peripheral tissues and cells also likely play a crucial role in keeping retina health. As reviewed by Wei et al., alteration of the expression profile and function of miRNAs induces a dysfunction of immune system leading to autoimmune disorders, which in part affect the eye. In this review, Wey et al. highlight the roles of miRNAs as main actors implicated in most representative ocular autoimmune disorders, including autoimmune uveitis, Grave's ophthalmopathy, and Sjögren's syndrome dry eye. Importantly, they discuss the potential of circulating miRNAs as biomarkers for autoimmune-mediated eye disorders, along with a future exploration of miRNA-based therapeutic approaches.

Intartaglia et al. assessed the contribution of the emerging roles of miRNAs in the function and health of the Retinal Pigment Epithelial (RPE) cells and on the future exploration of miRNA-based therapeutic approaches to counteract blinding diseases. More specifically, they highlight the crosstalk between miRNAs function and circadian regulation of phagocytosis and POS cell clearance as active part in the visual cycle and function.

In addition, six original research papers also provided evidence on the role of miRNAs involved in regulating the visual system. Fernando et al. explored the molecular pathways in miR-223 KO mice demonstrating the functional role of this miRNA in controlling inflammation in retinal and circulating immune cells during retinal degeneration. They highlight the relevance of miR-223 in physiological normal conditions and in a photo-oxidative damage model of retinal degeneration, discussing future perspectives on the relevance in defining the therapeutic utility to modulate miR-223 expression and function in retinal degenerative diseases.

Interestingly, Chen et al. reported the retinal consequences of loss of function of the most abundant circular RNA, namely Cdr1as. They carried out an extensive study demonstrating that the abundance Cdr1as is required for retinal development and maintenance and discussing future perspectives on the role of this circRNA in the visual system. In this topic focusing on the role of ncRNAs involved in regulating the differentiation and maintenance of fovea in primate's retina, Fishman et al. characterized the transcriptional landscape of the developing rhesus monkey retina and found differentially expressed miRNAs during fovea development. Notably, this study not only provides novel datasets for a more comprehensive understanding of fovea formation and function, but it led to hypothesize that dysfunction of specific miRNA could contribute to fovea disease, such as age-related degeneration and noise- or toxic fovea damage. A pioneer study aimed at defining the function of miRNAs in the in reactive Müller Glia cells was carried out by Kang et al., through the conditional inactivation of Dicer in Muller cells profiled both miRNAs and mRNAs in reactive Müller Glia cells after light damage. This study points to miRNA-based therapeutic approaches to attenuate gliosis. The study by Xu et al. provided compelling evidence for the involvement of miR-183/96/182 cluster in human cause IRD. They carried out a mutational screening that took into account the three members of the miR-183 cluster and identified six sequence variants in a large cohort of patients, three in pre-miR-182 and three in pre-miR-96 and highlighted the potential roles of these miRNAs in the susceptibility to IRDs. Finally, along a clinical investigation in identifying pathophysiological roles of ncRNAs in ocular tissues and visual function, Anand et al. constructed the miRNAs-mRNA gene regulatory networks downstream to Tdrd7, a Tudor domain protein involved in post transcription regulation of gene expression and mutated in congenital cataracts. The comprehensive dataset of miRNAs and mRNA identified in the study point to novel pathways involved in maintaining lens function and homeostasis throughout life.

To conclude, this Research Topic summarized the current information on the relevance of ncRNAs in visual system. The number and selection of papers considered within this Research Topic demonstrate the complex, multilevel activity of ncRNAs in the pathogenesis of both monogenic and complex genetic disorders and summarizes the current effort to improve diagnosis and develop treatments to eye pathologies.

## Author Contributions

All authors have contributed to write the Editorial manuscript and approved the submitted version.

## Conflict of Interest

The authors declare that the research was conducted in the absence of any commercial or financial relationships that could be construed as a potential conflict of interest.

## Publisher's Note

All claims expressed in this article are solely those of the authors and do not necessarily represent those of their affiliated organizations, or those of the publisher, the editors and the reviewers. Any product that may be evaluated in this article, or claim that may be made by its manufacturer, is not guaranteed or endorsed by the publisher.

